# The Hidden Cost of Bariatric Surgery: Wernicke’s Encephalopathy and Polyneuropathy

**DOI:** 10.7759/cureus.81337

**Published:** 2025-03-28

**Authors:** Lailus Sabikunnahar Mishu, Esraa Abuelgassem Hagahmed Mohamed, Tochukwu Samuel Odogwu, Muhammad Aftab Toor, NoimUddin Jibon

**Affiliations:** 1 Respiratory Medicine, Mid and South Essex NHS Foundation Trust, Southend-On-Sea, GBR; 2 Internal Medicine, Mid and South Essex NHS Foundation Trust, Southend-On-Sea, GBR; 3 Medicine, Mid and South Essex NHS Foundation Trust, Southend-On-Sea, GBR; 4 Medicine, Mid and South Essex NHS Foundation Trust, Southend-on-Sea, GBR

**Keywords:** bariatric surgery, bariatric surgery complications, polyneuropathy, thiamine deficiency, wernicke's encephalopathy

## Abstract

Bariatric surgery is a recognised intervention for severe obesity, a global epidemic associated with numerous comorbidities like diabetes, hypertension, and cardiovascular diseases. Procedures such as Roux-en-Y gastric bypass facilitate significant weight loss, improving metabolic health and quality of life. However, these surgeries carry hidden costs, particularly concerning nutritional deficiencies that are often overlooked in preoperative assessments.

This case report presents a 27-year-old patient who, after undergoing gastric sleeve surgery, developed neurological symptoms due to thiamine deficiency. Thiamine, essential for energy metabolism, is crucial for neurological function, and its deficiency can lead to serious conditions like Wernicke’s encephalopathy and polyneuropathy. Despite the initial success of the surgery, the patient faced significant morbidity due to this oversight.

The report emphasises the need for rigorous nutritional monitoring and proactive supplementation post-surgery. It advocates for comprehensive preoperative counselling that addresses potential deficiencies and their long-term implications. By recognising these hidden costs, healthcare providers can better prepare patients for effective long-term management, enhancing post-operative outcomes and improving the overall quality of life for those battling severe obesity.

## Introduction

Obesity is a significant global health challenge, contributing to chronic conditions such as cardiovascular disease, diabetes, and obstructive sleep apnoea (OSA), which increase morbidity and mortality [1]. For severe obesity (BMI ≥ 40 kg/m² or ≥35 kg/m² with comorbidities), bariatric surgery remains the most effective treatment for sustained weight loss [2]. Sleeve gastrectomy (SG), a widely performed procedure, reduces gastric volume and alters gut hormone secretion, though its anatomical changes also predispose patients to micronutrient deficiencies [3].

Among these deficiencies, thiamine (vitamin B1) deficiency is particularly consequential, as it can lead to Wernicke’s encephalopathy (WE) and polyneuropathy-neurological emergencies often underrecognised in post-bariatric patients [4]. WE classically presents with confusion, ataxia, and ophthalmoplegia (though the full triad is rare), and untreated cases may progress to irreversible Korsakoff syndrome. This risk is exacerbated in settings with poor post-operative monitoring, such as medical tourism, where adherence to supplementation guidelines is inconsistent [5].

While guidelines (e.g., American Society for Metabolic and Bariatric Surgery (ASMBS)) recommend lifelong thiamine supplementation (≥12 mg/day) and multidisciplinary follow-up [4,5], cases of WE persist due to delayed diagnosis or inadequate care. We present a case highlighting this preventable complication, emphasising the need for heightened clinical vigilance and patient education post-SG.

## Case presentation

A 27-year-old lady underwent gastric sleeve surgery abroad in February 2024 for the management of morbid obesity. The surgery was performed outside the United Kingdom, and the patient had no known history of nutritional deficiencies. She was not taking any nutritional supplements prior to the procedure. However, details of her preoperative nutritional screening were unavailable, as the evaluation was conducted abroad, and she was not enrolled in any post-operative follow-up programme. Within three months of the procedure, the patient presented to the Emergency Department with recurrent falls and progressive bilateral lower limb weakness. The symptoms initially manifested as weakness and paraesthesia in the feet, which gradually ascended proximally, leading to impaired ambulation. She was initially diagnosed with acute neuropathy with demyelination (Guillain Barre Syndrome) following review by neurologists. She was managed with intravenous immunoglobulins while on admission to the acute medical unit.

Initial laboratory investigations revealed a haemoglobin level of 137 g/L, red cell distribution width (RDW) of 18.0, and a white blood cell count of 7.3 × 10⁹/L, with normal neutrophil and lymphocyte counts. A venous blood gas showed a normal level of bicarbonate and lactate. Cerebrospinal fluid (CSF) analysis demonstrated clear, colourless fluid with a white cell count of 12, total protein of 0.40 g/L, and glucose level of 3.8 mmol/L. No organisms were identified on culture. Extensive viral PCR testing was negative for herpes simplex virus types I and II, varicella-zoster virus, enterovirus, and parechovirus. Serum vitamin B12 level was elevated at 732 pmol/L, and folate was within normal limits at 8.1 nmol/L. Myeloma screening performed during the admission yielded normal results.

**Figure 1:**
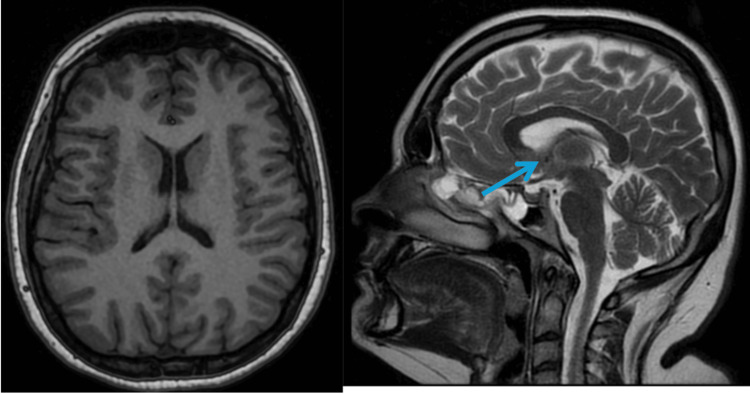
MRI Brain - Cross-sectional & Sagittal Views Reported as normal.

**Figure 2:**
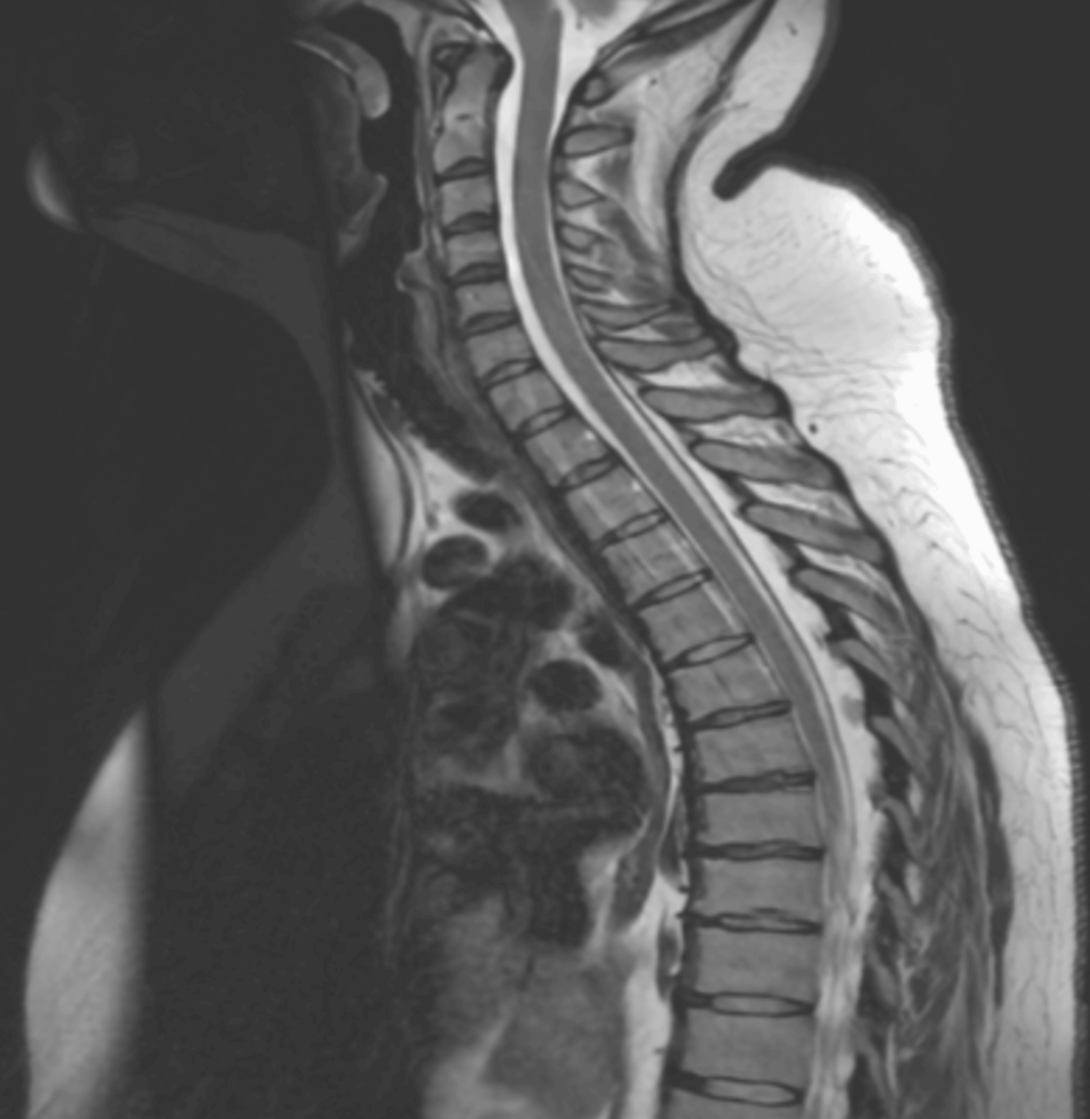
MRI Cervical and Thoracic Spines. Reported as normal.

**Figure 3:**
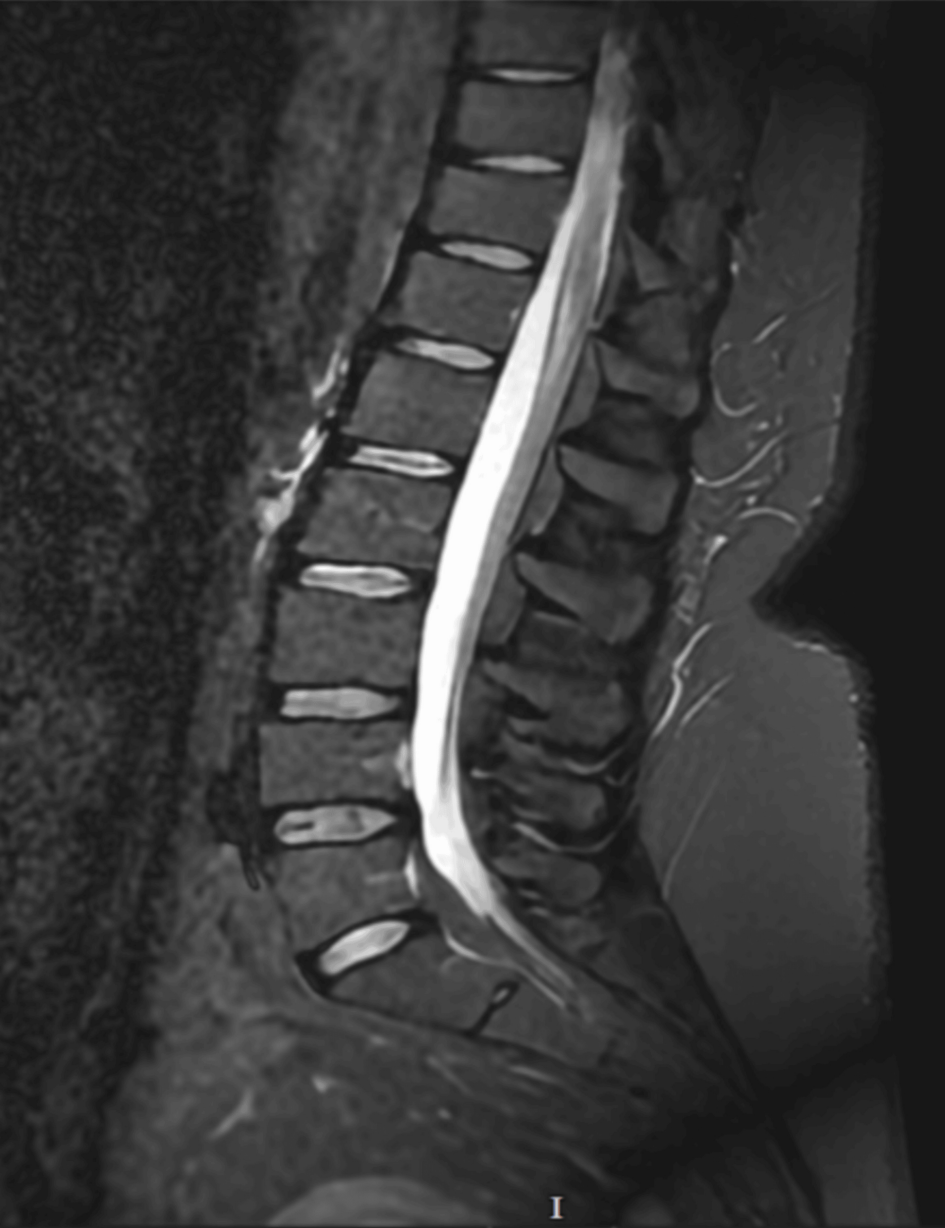
MRI of Lumbosacral Spine Showing Mild Diffuse Disc Bulging of L4/L5 Without Significant Neural Compression

MRI head and whole spine in Figures 1-3 was done to rule out intracranial or spinal pathologies. The only abnormality noted was mild diffuse disc bulging at L4/L5 Figure 3 without any significant neural compression. She was discharged after 7 days in the AMU with outpatient follow-up, electromyography (EMG) and nerve conduction studies appointments. The patient re-presented to the Emergency Department within one week with new-onset ophthalmoplegia and diplopia, along with worsening lower limb symptoms that had progressed to include upper limb numbness and weakness. On admission, she also exhibited diaphragmatic weakness, with type-2 respiratory failure on arterial blood gas (ABG) necessitating non-invasive ventilation (NIV) due to a reduced vital capacity of 1.2 L, though this later improved. MRI of the brain in Figure 1 was reported as normal, though there was some debate regarding a subtle abnormality in the mammillary bodies, which favours the diagnosis of WE. MRI of the spinal cord was unremarkable in Figures 2-3. EMG revealed a severe axonal neuropathy with distal predominance in Tables 1-4 and sleep study ruled out any OSA. Based on these findings, she was diagnosed with WE and associated polyneuropathy due to thiamine deficiency. She received aggressive treatment with intravenous Pabrinex over the course of one month. A blood sample was collected later for thiamine level, after the intensive thiamine therapy, and the result was normal - 292. A formal cognitive assessment identified deficits in memory, though these have gradually improved over time, as has her ataxia. She was subsequently transferred to specialist Inpatient neurological rehabilitation.

**Table 1:** Sensory Nerve Conduction NR: No response is detected; ms: milliseconds; m/s: metres per second; mm: millimetres; R: right; L: left; μv: microvolt

Nerve and Site	Onset Latency	Peak Latency	Amplitude	Segment	Latency Difference	Distance	Conduction Velocity
Median - R							
Dig-II	NR	NR	NR	Dig-II-Wrist	ms	mm	m/s
Dig-III	NR	NR	NR	Dig-III-Wrist	ms	mm	m/s
Ulnar - R							
Dig V	NR	NR	NR	Dig-V-Wrist	ms	mm	m/s
Radial - R							
Forearm	NR	NR	NR	Forearm-wrist	ms	mm	m/s
Radial - L							
Forearm	NR	NR	NR	Forearm-wrist	ms	mm	m/s
Median - R							
Wrist	NR	NR	NR	Wrist-Dig-II	ms	mm	m/s
Ulnar - R							
Wrist	1.9 ms	2.5 ms	14 μv	Wrist-Dig-V	1.9 ms	100 mm	54 m/s
Sural - R							
Calf	NR	NR	NR	Calf-ankle	ms	mm	m/s
Superficial peroneal - R							
Calf	NR	NR	NR	Calf-ankle	ms	mm	m/s
Sural - L							
Calf	NR	NR	NR	Calf-ankle	ms	mm	m/s
Superficial peroneal - L							
Calf	NR	NR	NR	Calf-ankle	ms	mm	m/s

**Table 2:** Motor Nerve Conduction All recorded motor responses are either undetectable or low-amplitude NR: no response; ms: milliseconds; mm: millimetres; m/s: metres per second; mV: millivolt; R: right; L: left

Nerve and Site	Latency	Amplitude	Segment	Latency Difference	Distance	Conduction Velocity
Median - R						
Wrist	NR	NR	Abductor pollicis brevis-wrist	ms	mm	m/s
Ulnar - R						
Wrist	2.3 ms	5.7 mV	Wrist-Abductor digiti minimi	2.3 ms	mm	m/s
Below elbow	6.9 ms	5.0 mV	Wrist-Below elbow	4.6 ms	210 mm	46 m/s
Above elbow	8.3 ms	3.9 mV	Below elbow-Above elbow	1.4 ms	60 mm	43 m/s
Median - L						
Wrist	NR	NR	Abductor pollicis brevis-wrist	ms	mm	m/s
Ulnar - L						
Wrist	3.3ms	0.3mV	Wrist-Abductor Digiti minimi	3.3 ms	mm	m/s
Peroneal - R						
Ankle	NR	NR	Ankle-Extensor digitorum brevis	ms	mm	m/s
Peroneal - L						
Ankle	NR	NR	Ankle-Extensor digitorum brevis	ms	mm	m/s
Tibial - R						
Ankle	5.0 ms	0.2 mV	Ankle-Abductor hallucis	5.0 ms	mm	m/s
Tibial - L						
Ankle	4.7 ms	0.1 mV	Ankle-Abductor hallucis	4.7 ms	mm	m/s

**Table 3:** F-Wave Studies Prolonged right ulnar F wave. No F wave is recorded from the right and left tibial nerves.

Nerve	M-Latency	F-Latency
Ulnar - R	2.3	30.4

**Table 4:** Needle EMG Examination No Vol MU: No Voluntary Motor Units; Max.: Maximum; R: right; L: left; EMG: electromyography

	Insertional	Spontaneous and/or Volitional Activity						Maximum Volitional Activity		
Muscle	Insertional	Fibs	+Wave	Fasc	Duration	Amplitude	Poly	Amplitude	Pattern	Effort
Tibialis anterior - R	Increased	4+	4+	None	No Vol MU	No Vol MU	None	No Vol MU	None	Max.
Vastus lateralis - R	Increased	4+	4+	None	No Vol MU	No Vol MU	None	Normal	None	Max.
Gastrocnemius - R	Increased	4+	4+	None	Normal	Normal	None	Normal	Single unit	Max.
Tibialis anterior - L	Increased	4+	4+	None	No Vol MU	No Vol MU	None	No Vol MU	Single unit	Max.
Vastus lateralis - L	Increased	4+	4+	None	No Vol MU	No Vol MU	None	No Vol MU	None	Max.
1^st^ dorsal interosseous - R	Normal	None	None	None	Normal		None	Normal	Reduced	Max.
Extensor digitorum communis - R	Increased	4+	4+	None	No Vol MU	No Vol MU	None	No Vol MU	None	Max.
Deltoid - R	Increased	None	None	None	Normal	Normal	None	Normal	Full	Max.
Extensor digitorum communis - L	Increased	4+	4+	None	No Vol MU	No Vol MU	None	No Vol MU	None	Max.

However, she continues to experience significant motor and sensory neuropathy, with persistent marked weakness, particularly affecting her hands and wrists. Given the severity of her neuropathy, clinical recovery is expected to be slow and prolonged.

## Discussion

Thiamine (vitamin B1) deficiency, though rare in the general population, is a well-documented complication following bariatric surgery, with prevalence rates ranging from 18% to 49% in post-operative patients [6]. This case highlights the critical importance of recognising and addressing thiamine deficiency early to prevent severe neurological sequelae such as WE and polyneuropathy.

WE is a medical emergency and the complete triad is present in only about 16% of cases [7]. In this patient, the initial presentation of recurrent falls, bilateral lower limb weakness, and subsequent development of ophthalmoplegia and diplopia were indicative of WE, though the diagnosis was initially missed. This delay in diagnosis is not uncommon, as the symptoms of WE can be nonspecific and overlap with other neurological conditions, such as Guillain-Barré syndrome, which was initially suspected in this case. The prevalence of WE following bariatric surgery is estimated to be between 0.2% and 2%, though this may be an underestimation due to under-diagnosis and misdiagnosis [6].

Thiamine deficiency in bariatric surgery patients stems from malabsorption due to anatomical changes, post-operative vomiting, reduced intake, and poor supplementation adherence [8, 9]. In this case, SG increased deficiency risks due to reduced food intake and malabsorption. The patient’s rapid progression to severe neurological symptoms within three months post-surgery underscores the need for vigilant monitoring and early intervention.

The management of WE requires aggressive thiamine replacement therapy, typically administered intravenously in the form of Pabrinex, as was done in this case. Early treatment is crucial to prevent the progression to Korsakoff syndrome, a chronic and often irreversible condition characterised by severe memory impairment and confabulation [7]. In this patient, the initiation of intravenous thiamine led to gradual improvement in cognitive function and ataxia, though significant motor and sensory neuropathy persisted. This underscores the importance of early recognition and treatment, as delayed intervention can result in long-term neurological deficits.

The prevalence of polyneuropathy following bariatric surgery is less well-documented, though it is recognised as a potential complication of thiamine deficiency. In this case, the patient developed severe axonal neuropathy with distal predominance, which contributed to her persistent weakness and sensory deficits. The slow and prolonged recovery expected in this patient is consistent with the natural history of axonal neuropathy, which often requires months to years for significant improvement, if at all [6]. This highlights the need for long-term neurological rehabilitation and support for patients who develop such complications.

## Conclusions

In conclusion, this case highlights the hidden costs of bariatric surgery, particularly the risk of nutritional deficiencies and their severe neurological consequences. Thiamine deficiency, though preventable, remains a significant concern in post-bariatric surgery patients, with potentially devastating outcomes if not promptly recognised and treated. The prevalence of WE and polyneuropathy following bariatric surgery underscores the need for rigorous nutritional monitoring, proactive supplementation, and comprehensive patient education. By addressing these issues, healthcare providers can improve post-operative outcomes and enhance the quality of life for patients undergoing bariatric surgery.
